# Doubling the known endemic species diversity of New Caledonian armored scale insects (Hemiptera, Diaspididae)

**DOI:** 10.3897/zookeys.782.27938

**Published:** 2018-08-16

**Authors:** Nate B. Hardy, Douglas J. Williams

**Affiliations:** 1 Department of Entomology and Plant Pathology, Auburn University, 301 Funchess Hall, Auburn, Alabama 36849, USA Auburn University Auburn United States of America; 2 Department of Life Sciences, The Natural History Museum, London, UK Department of Life Sciences, The Natural History Museum London United Kingdom

**Keywords:** biodiversity hotspot, taxonomy, southern hemisphere

## Abstract

Fourteen species of armored scale insects are known only from New Caledonia. Here, the adult female of fourteen more are described: *Agrophaspisansevatae***sp. n.**, *Aonidiamontikoghis****sp. n.***, *Aonidiapauca***sp. n.**, *Fernaldannawhita***sp. n.**, *Furcaspiscostulariae***sp. n.**, *Greeniellacasuarinae***sp. n.**, *Greenielladacrydiae***sp. n.**, *Lepidosaphesmonticola***sp. n.**, *Leptaspispege***gen. et sp. n.**, *Leucaspismontikoghis***sp. n.**, *Melanaspisnothofagi***sp. n.**, *Neomorganianothofagi***sp. n.**, *Pseudaonidiadugdali***sp. n.**, and *Pseudaonidiayateensis***sp. n.** We note that the diversity of New Caledonian armored scale insects appears to have resulted more from trans-oceanic dispersal than in situ speciation.

## Introduction

New Caledonia is a biodiversity hotspot. Before 80 Ma, it was part of the southern super-continent Gondwana, and much of its current biota is phylogenetically related to lineages inhabiting other Southern Hemisphere landmasses. But in the Oligocene, ~37 Ma ago, New Caledonia was completely submerged in the South Pacific (Grandcolas et al. 2008). Therefore, the terrestrial species that live there today are presumably descended from ancestors that managed to make it across an ocean and establish a new New Caledonian population. Since its reemergence, how many times has New Caledonia been colonized? How much of its biota is the product of in situ diversification? How did it come to be so diverse so rapidly? Here we add to our store of the basic information that we will need to answer these questions – information about what species actually occur in New Caledonia. Specifically, we describe fourteen new species of armored scale insects endemic to New Caledonia.

Prior to this work, 56 species of armored scale insects had been recorded from New Caledonia (García Morales et al. 2016, Mille et al. 2016, Hamilton et al. 2017). Only 14 of these, including five species of *Andaspis* described recently (Hamilton et al. 2017) are known only from New Caledonia. With the exception of a handful of species that are also found in a few other locations in the South Pacific, the other armored scale insects in New Caledonia are polyphagous, cosmopolitan pest species. Thus, the fourteen species we describe here increases the total species richness by 25%, and the endemic species richness by 100%.

## Materials and methods

This work is mostly based on specimens that were collected from New Caledonia 40–50 years ago, and were slide-mounted and deposited in the Natural History Museum, London, UK (**NHMUK**) or the US National Scale Insect Collection in Beltsville, Maryland (**USNM**). The number of specimens examined for each species is provided at the beginning of each description. When a sufficient number of specimens were in hand, we deposited paratypes in the NHMUK, USNM, and the Muséum national d’Histoire naturelle in Paris, Franc (**MNHN**). For each species, we mostly describe just one side of the body, including the mid line. This avoids the ambiguity that can arise when reporting the numbers of structures that occur on each side of a bilaterally symmetrical animal. It also spares us the repetition of chasing each count with the qualifier that it applies to each side of the body. Nevertheless, in some cases, a description of both sides of the body is easier to comprehend, and we eschew the efficiency of one-sided description in favor of enhanced comprehension. Specimens were viewed under 100–400× magnification, and phase contrast, with a Nikon Ni-E light microscope equipped with a Z-axis-motorized stage. Digital images and measurements were performed with the aid of NIS elements software. Digital images were focus-stacked with Zerene Stacker (Zerene Systems LLC). All measurements are maximum dimensions. The morphological terminology follows Williams and Watson (1988). Digital images were used as the starting point for line drawings. Following the convention for scale insects, each illustration shows the dorsal body surface on the left side, and the ventral body surface on the right side. We refer to pygidial lobes with a shorthand notation, combining the letter L with an index that increments from posteromedial to anterolateral. For example, on each side of the body, the medial-most lobe is referred to as L1, and the lobe immediately anterolaterad of it is referred to as L2.

## Taxonomy

### Agrophaspis ansevatae sp. n.

Taxon classificationAnimaliaHemipteraDiaspididae

http://zoobank.org/53E449E7-B273-4070-B311-1059062CDFC1

Figure 1

#### Material examined.

*Holotype*: New Caledonia: 1 adult female (0.49 mm long, 0.46 mm wide): *ex* undetermined tree, shore south of Anse Vata, Noumea, 17.viii.1963, leg. SW Brown, SWB accession 252 (USNM). *Paratypes*: New Caledonia: 1 adult female, same data as holotype; exuviae of 1 second-instar: on same slide as holotype, SWB accession 252 (USNM).

#### Description.

**Adult female, n = 2.** Pupillarial. Body 0.38–0.49 mm long, broadest at anterior abdominal segments (0.36–0.46 mm); outline roughly triangular, posterior margin truncate, thorax and head tapering anteriorly.

*Pygidium* truncate, with four long tapering caudal projections, lacking typical lobes and plates. Dorsum with sclerotic patches around and behind anus. Anus circular, near center of pygidium. Venter of pygidium with vulva near center, about as far from posterior margin as anus. No perivulvar pores. Microducts scattered along margin, at least one near base of each caudal projection.

*Prepygidial segments* Venter with microducts along margin of abdominal segments; small setae in submedial and marginal areas of abdomen. No pores present near spiracles. Antennae each with three fleshy setae.

**Puparium (cuticle of second-instar female)** (not illustrated). *Pygidium* with three pairs of lobes, medial lobes each with lateral and medial notch, second and third lobes each with lateral notch; fringed plates between lobes. Macroducts one-barred. Anus near posterior margin, diameter less than width of medial lobe. Margin with many gland tubercles.

**Figure 1.**
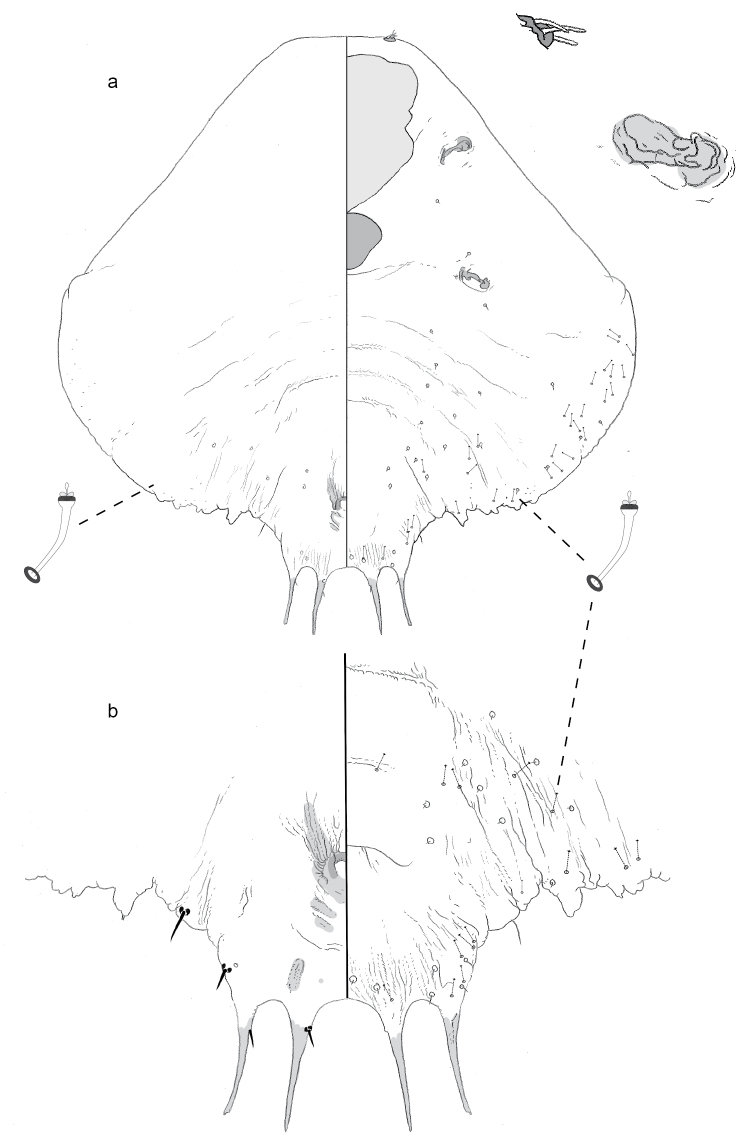
**a** Adult female of *Agrophaspisansevatae* sp. n. with **b** enlargement of pygidium.

#### Comments.

Borchsenius and Williams (1963) erected the genus *Agrophaspis* by monotypy for the New Caledonian pupillarial species *Aonidiabuxtoni* Laing. The adult female of that species shares several traits with the adult of *A.ansevatae*; for example, both lack perivulvar and spiracular pores. Most strikingly, they both lack typical lobes and plates on the pygidium, and have in their place long, tapering caudal projections. In *A.buxtoni* there are seven, and each is bifid or trifid. In *A.ansevatae* there are four. On the holotype, at least one of these also appears to have a slightly bifid apex. The puparia are also similar, but in *A.ansevatae* the diameter of the anus is less than the width of L1, whereas in *A.buxtoni* it is greater than the width of L1.

#### Etymology.

The species epithet is taken from the specimens’ provenance, near Ansa Vata.

### Aonidia montikoghis sp. n.

Taxon classificationAnimaliaHemipteraDiaspididae

http://zoobank.org/821958F8-75E4-4DA3-9E25-E76087B4DC8D

Figure 2a, b

#### Material examined.

*Holotype*: New Caledonia: 1 adult female (0.49 mm long, 0.28 mm wide): *ex* ?*Metrosideros* sp., Mt. Koghia [sic], 5.x.1978, leg JS Dugdale, BM 19 13 (NHMUK). *Paratypes*: New Caledonia: 3 adult females and exuviae of 3 second-instars (i.e., puparia) on five slides: same data as holotype, BM 19 13 (NHMUK, USNM, MNHN).

#### Description.

**Adult female, n = 4.** Pupillarial. Body 0.48–0.51 mm long, broadest at anterior abdominal segments (0.28–0.31 mm); outline roughly fusiform, posterior margin truncate.

*Pygidium* without differentiated lobes. Dorsum of pygidium becoming more sclerotic from anterior to posterior end, membranous patches of cuticle in anterior half, narrow linear furrows of membranous cuticle near and perpendicular to posterior margin. Anus circular (~ 11 μm in diameter), near anterior edge of the pygidium. No ducts detected. Venter of pygidium with vulva in anterior half. No perivulvar pores. A few setae scatted along dorsal and ventral submargin and medial areas.

*Prepygidial segments* Dorsum with fine, hair-like setae, scattered along margin, few also present on medial areas of abdomen. Ducts absent. On venter, small setae in loose longitudinal submedial and submarginal lines across abdominal segments. No ducts or pores present. Antennae each with two fleshy setae. No pores present near spiracles.

**Figure 2.**
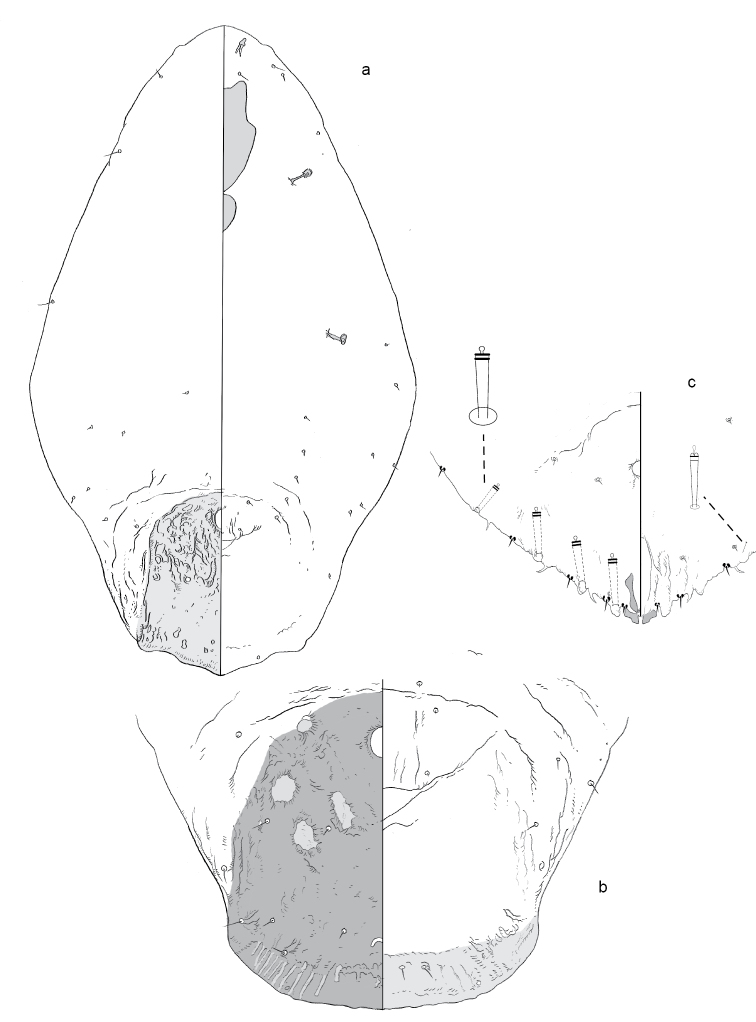
**a** Adult female of *Aonidiamontikoghis* sp. n. with enlargements of pygidium of **b** adult and **c** 2^nd^-instar.

**Puparium (cuticle of second-instar female)** (Figure 2c). *Pygidium* with only medial lobes, each with lateral notch on apex. Anus circular in anterior half of pygidium. Two-barred macroducts on margin, four on each side, posterior three ducts stemming from distinct pore prominence. A basal sclerosis extending from inner edge of each L1 on each side of body, converging medially to form a triangular carina. One simple gland spine just mesal of second pore prominence, and another just mesal of the third. Few microducts present in submarginal area.

#### Comments.

The adult female of *A.montikoghis* shows little that can be used to make a generic assignment. The second-instar female / puparium is of more use. The pygidium of the second-instar female is most similar to that of the Australian species *Alioidestuberculatus* (Laing). That also has (1) a triangular carina diverging from the inner edges of the medial lobes, (2) only the medial pygidial lobes present, (3) two-barred marginal macroducts, each stemming from a distinct pore process, and (4) no other dorsal macroducts on the pygidium (Brimblecombe 1958). The adult female of *A.tuberculatus* is not pupillarial, but unpublished DNA-sequenced based phlogeny estimates recover *Alioides* nested within the pupillarial genus *Aonidia* (B. Normark pers. comm.). Thus, *Aonidia* seems to be the best fit for this species.

#### Etymology.

The species epithet is taken from the specimens’ provenance, Mount Koghis. It is also meant to reflect that this species, like the type species, is known from a mountain on an island. The name is a noun in apposition.

### Aonidia pauca sp. n.

Taxon classificationAnimaliaHemipteraDiaspididae

http://zoobank.org/49478016-4BCD-465A-A094-6D4BCFE06295

Figures 3 , 4

#### Material examined.

*Holotype*: 1 adult female (0.34 mm long, 0.23 mm wide), ex myrtaceous shrub, Mont D’Or, roadside fountain, 24.viii.1963, leg. SW Brown, SWB accession 260 (USNM). *Paratypes*: New Caledonia: 3 adult females (1 in mounting medium just outside of cover slip, on right side of slide), 1 second-instar, exuviae of 2 second-instars (i.e., puparia), and exuviae of two first-instars: *ex* unknown host, Yaté, 10.iv.2014, leg. S Cazères, 137-14, COCHE/34/14 (NHMUK).

#### Description.

**Adult female, n = 3.** Pupillarial. Body 0.34–0.50 mm long, broadest at metathorax (0.18–0.27 mm); outline roughly fusiform, posterior margin truncate.

*Pygidium* with 3–4 differentiated lobes on each side, but no plates or gland spines. Dorsum of pygidium more sclerotic on posterior than anterior end, membranous patches of cuticle in anterior half. Anus circular (~11 μm in diameter), near anterior edge of the pygidium. Microducts scattered along submargin. No macroducts detected. Venter of pygidium with vulva in anterior half. No perivulvar pores. Distinct transverse ridge present anterior of lobes.

*Prepygidial segments* Dorsum with fine, hair-like setae, scattered along margin. Ducts absent. Conspicuous tubercle on each side of head. On venter, small setae in loose longitudinal submedial and submarginal lines across abdominal segments. A few microducts in marginal areas of abdominal segments. Each anterior spiracle with a cluster of three trilocular pores. Antennae each with two fleshy setae.

**Second-instar female.***Pygidium* with only medial lobes, each with lateral notch. Anus circular in anterior half of pygidium. Large two-barred macroducts on margin, four on each side, posterior three ducts opening into distinct pore prominence. A basal sclerosis extending from inner edge of each L1 on each side of body, converging medially to form a triangular carina. One simple gland spine just mesal of each pore prominence. A few microducts present in submargrinal area.

**Figure 3.**
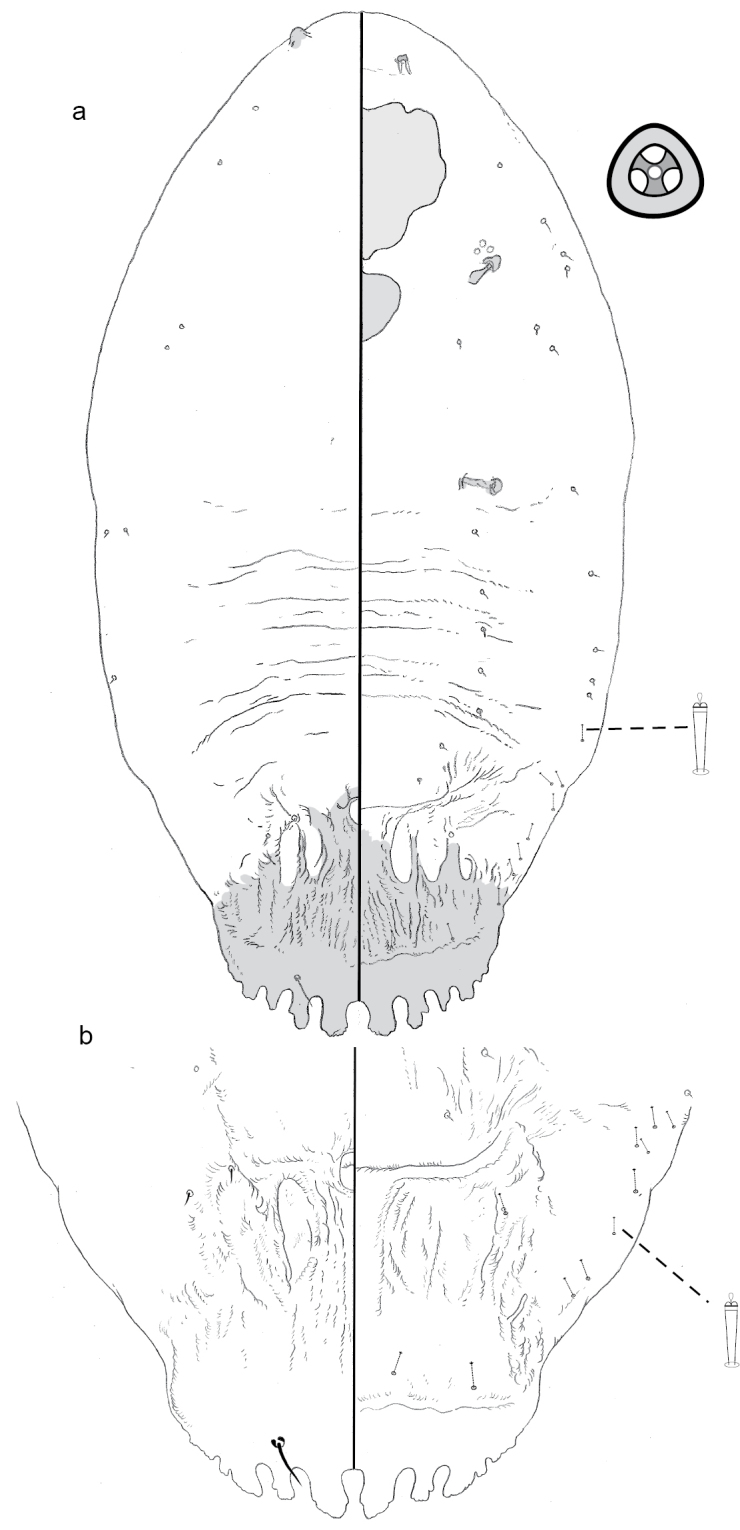
**a** Adult female of *Aonidiapauca* sp. n. with **b** enlargement of pygidium.

**Figure 4.**
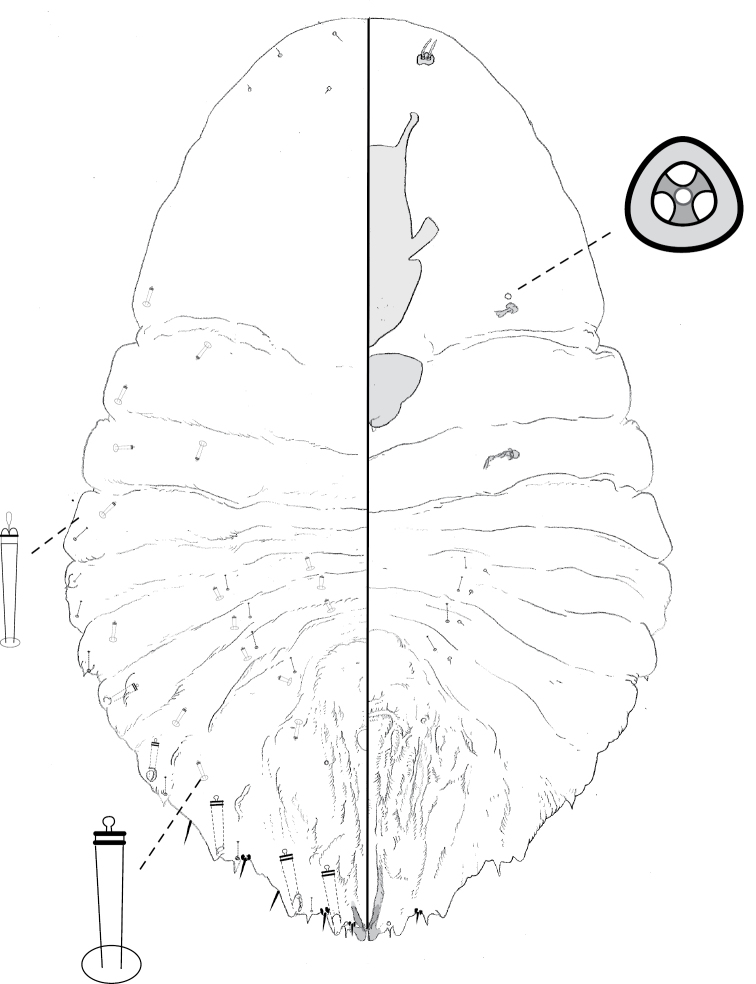
Second instar of *Aonidiapauca*.

#### Comments.

Like the adult female of *A.montikoghis*, that of *A.pauca* is bereft of many diagnostic characters. It can be easily distinguished from the former by having well-developed pygidial lobes and tilocular pores near the anterior spiracles.

#### Etymology.

The specific name *pauca* is the Latin feminine adjective *paucus*, meaning few and referring to the simplicity of the morphology of the adult female.

### Fernaldanna whita sp. n.

Taxon classificationAnimaliaHemipteraDiaspididae

http://zoobank.org/7947E043-AB40-498D-A6B5-134FF317825B

Figure 5

#### Material examined.

*Holotype*: New Caledonia: 1 adult female (0.84 mm long, 0.28 mm wide): *ex* undetermined host, Whita River area, 5.ix.1963, SW Brown, SWB accession 270 (USNM).

#### Description.

**Adult female, n = 1.** Body of holotype 0.84 mm long, broadest at metathorax (0.28 mm); body outline elongate.

*Pygidium* longer than wide; with two distinct lobes on each side. Median lobes wider than long, each with slightly rounded apex. L2 much wider (~2×) than L1. Pygidial margin anterior to L2 serrate, with narrow sclerotic straps. Distinct paraphyses not discerned. No plates between median lobes or between L1 and L2; one fringed plate lateral of L2. Anus in anterior third of pygidium. Dorsal macroducts two-barred, restricted to margin and submargin; one arising from pore prominence adjacent to medial base of L2, one in pore furrow above lateral subunit of L2, seven anterior of L2. Dorsum with distinct patches of sclerotic cuticle, one large medial patch broadest just anterior of anus, tapering caudally; 2–3 marginal patches and one near anterolateral corner of medial patch. Venter of pygidium with vulva close to level of anus. Perivulvar pores ~5 μm in diameter, in two distinct groups (anteromedial and anterolateral groups confluent on holotype); ~10 in posterolateral group, ~10 anterolateral and anteromedial of vulva.

*Pre-pygidial segments.* Dorsum with few, fine, hair-like setae along anterior margin of head and a few on submargin and medial areas of thorax and anterior abdominal segments. On venter, small macroducts scattered along margin and submargin, few in medial areas of meso-, meta-thorax, and anterior abdominal segments. Abdomen with fine, minute setae on submedian and margin of segments just anterior of pygidium. Antenna with one long fleshy seta. Anterior spiracle with 2–3 quinquelocular pores. Posterior spiracle without pores.

**Figure 5.**
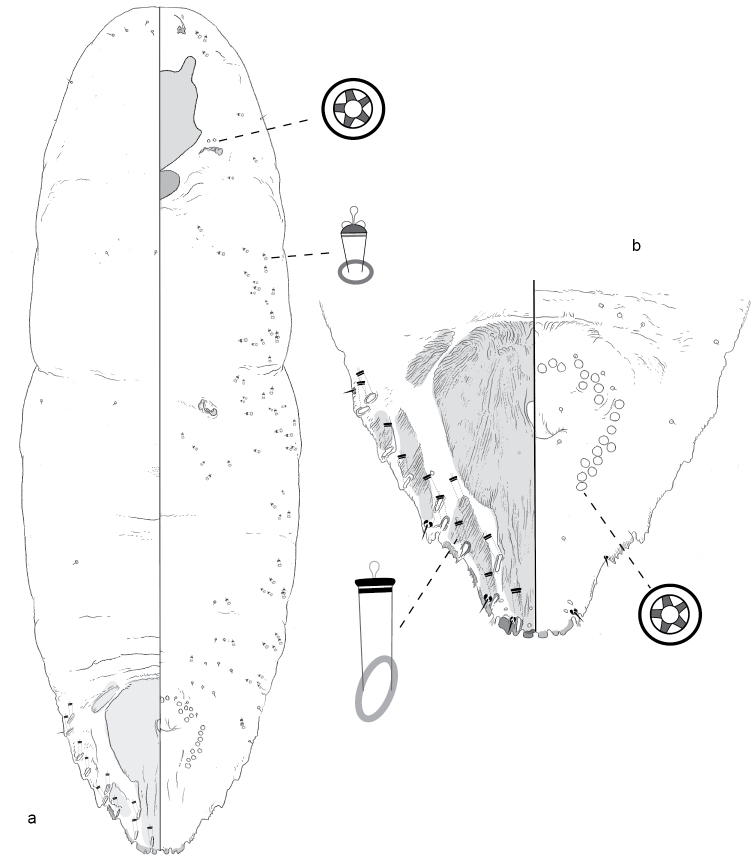
**a** Adult female of *Fernaldannawhita* sp. n. with **b** enlargement of pygidium.

#### Comments.

The genus *Fernaldanna* MacGillivrary (1921), previously contained only the type species *F.indentata* (Green), described from Australia on an unidentified host. The pygidium of the adult female of the type species shares several features with that of *F.whita*: (1) few or no plates; (2) two pairs of lobes, L1 with rounded apex, L2 broader than long and much broader than L1; (3) one marginal macroduct between L1 and L2, one at lateral base of L2, and a few more anterior. In *F.indentata* pygidial plates are completely absent; in *F.whita* there is just one plate laterad of L2. Both species have pores near the anterior spiracle, but only *F.whita* has pervivular pores. The adult female of *F.indentata* is pupillarial. We are not sure if the same is true of *F.whita*.

#### Etymology.

The species epithet is taken from its provenance, the Whita River area. It is a noun in apposition.

### Furcaspis costulariae sp. n.

Taxon classificationAnimaliaHemipteraDiaspididae

http://zoobank.org/192624C6-9DD1-483D-9BF1-88B2F6388D9E

Figure 6

#### Material examined.

*Holotype*: New Caledonia: 1 adult female (1.46 mm long, 1.23 mm wide): *ex Lophoschoenus* sp. [current valid name is *Costulariachamaedendron*], Mont d’Or, roadside fountain, 24.viii.1963, SW Brown, SWB accession 258 (USNM). *Paratype*: New Caledonia: 1 second-instar nymph, same data as holotype, SWB accession 258 (USNM).

#### Description.

**Adult female, n = 1.** Presumed to secrete scale cover. Body of holotype 1.46 mm long, broadest at mesothorax (1.23 mm); body outline turbinate (margin of head and thorax almost circular, abdomen tapering to truncate pygidium), margin of pre-pygidial abdominal segments slightly convex. Cuticle sclerotized (or at least heavily stained).

*Pygidium* wider than long, with three lobes on each side. Median lobes longer than wide, each with rounded apex, separated by ~2.5× their width. L2 and L3 as long as median lobe but broader, with more truncate apex. Pygidial margin anterior to L3 with a few long setae (up to 65 μm) and several tooth-like projections, pointed or truncate, smaller than lobes; posterior terminus of pygidium heavily sclerotized, distinct paraphyses not discerned. Plates each bifid, longer than lobes; two between median lobes, two between L1 and L2, 3 between L2 and L3, two anterior of L3. Anus in anterior third of pygidium, obstructed by detritus under cover-slip of slide mount. Thin one-barred macroducts abundant along posterior margin, ~18 on each side away from margin, some anterior of anus. Venter of pygidium with vulva in anterior half. Perivulvar pores absent.

*Pre-pygidial segments.* Dorsum with fine, hair-like setae along margin and submargin. Eye a subcircular disk on dorsal submargin lateral of antenna. Microducts scattered across head and thorax, along submarginal and submedial parts of abdominal segments grading into one-barred macroducts along margin of posterior pre-pygidial segments. On venter, microducts in loose transverse bands from submedian to margin of mesothorax and metathorax, posterior to each spiracle, also scattered along abdominal margin and submargin. Patches of gland tubercles on prothorax and mesothorax, 23–26 on each side of body, most on prothorax. Abdominal segments each with a small submedial seta with proportionately large, sclerotized collar, forming longitudinal rows from near lateral edge of vulva to posterior spiracles. Antennae each with four long setae. Anterior spiracle with cluster of nine quinquelocular pores. Posterior spiracle without pores.

**Figure 6.**
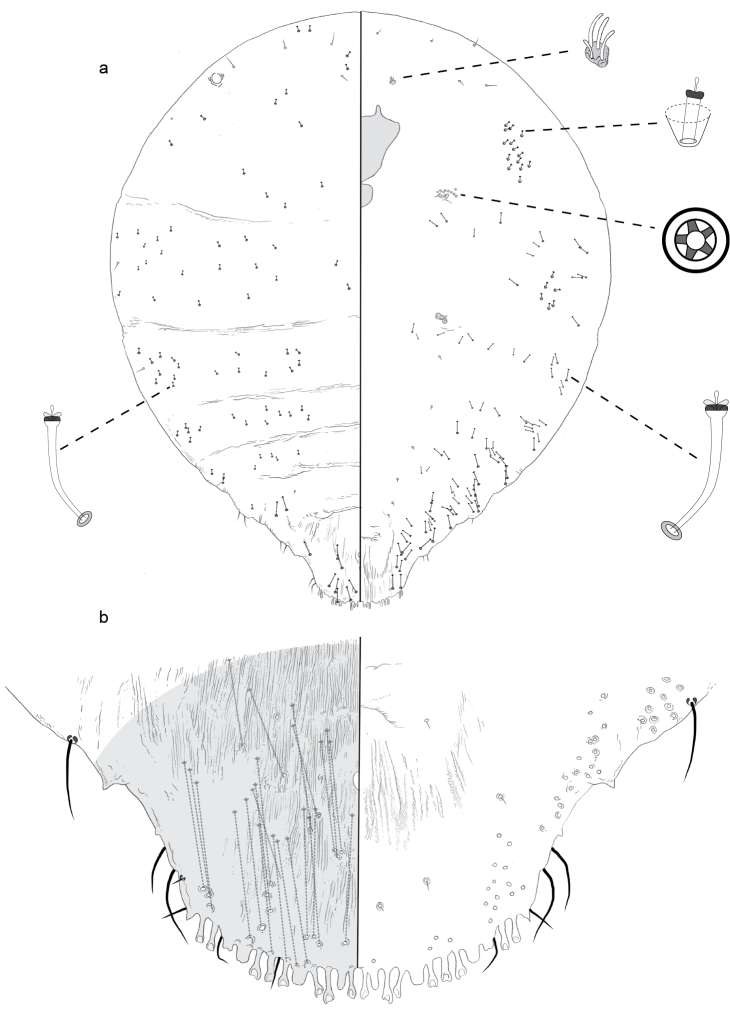
**a** Adult female of *Furcaspiscostulariae* sp. n. with **b** enlargement of pygidium.

#### Comments.

For a synthetic treatment of the genus *Furcaspis*, see the revision of Williams et al. (2006). Paraphrasing their diagnosis, adult females of *Furcaspis* have (1) simple bifurcate (rarely trifurcate) pygidial plates; (2) 3 pairs of notchless plates; (3) the antenna with multiple setae; (4) thin macroducts; (5) paraphyses; and (6) pores absent from the posterior spiracles. Currently, 29 species are recognized, two of which are endemic to New Caledonia: *F.cyphokantiae* Williams & Miller and *F.matileae* Williams & Miller. The adult female of *F.cyphokantiae* differs from that of *F.matileae* by having (1) gland tubercles on the venter of the mesothorax; (2) sclerotized lateral areas on the thorax; and (3) medial lobes that are closer together, and more similar in size and shape to the other lobes on the pygidium. The adult female of *F.costulariae* can be distinguished by having (1) only four long setae on the antenna (5–8, but usually six in *F.cyphokantiae* and *F.matileae*); (2) 23–26 gland tubercles, with only 2–3 on the mesothorax (tubercles absent from meso thorax, of *M.matileae*, and total 36–40 in *M.constulariae*); (3) uniformly sclerotic cuticle, and (4) lacking discernible paraphyses, although they may simply be impossible to detect against the background of sclerotic cuticle on the pygidial margin. All three New Caledonian species feed on monocotyledons; *F.cyphokantiae* and *F.costulariae* feed on sedges (Cyperaceae). It is possible that these three species are, in fact, just host-induced phenotypic variants of one New Caledonian metapopulation. Our decision to name the female from *Costularia* formalizes the alternative hypothesis that each host-associated form is a good species.

#### Etymology.

The species epithet is taken from the genus name of the host, a sedge that is endemic to New Caledonia.

### Greeniella casuarinae sp. n.

Taxon classificationAnimaliaHemipteraDiaspididae

http://zoobank.org/E2FCD996-4725-45DB-8996-F7DE71F58504

Figure 7

#### Material examined.

*Holotype*: New Caledonia: 1 adult female (0.43 mm long, 0.36 mm wide): *ex Casuarina* sp., near Yaté Dam, 3.ix.1963, SW Brown, SWB accession 267 (USNM).

#### Description.

**Adult female, n = 1.** Presumed pupillarial. Body of holotype 0.43 mm long, broadest at anterior abdominal segments (0.36 mm); body outline circular, with slight constriction at head.

*Pygidium* truncate, without dorsal macroducts, typical lobes, or plates; with six projections, each subtriangular, slightly bifid at apex. Anus circular, in posterior half of pygidium. Venter with vulva in posterior half, at level of anus. Perivulvar pores absent. Microducts scattered along posterior margin.

*Pre-pygidial segments.* Microducts scattered along margin of anterior abdominal segments, meta- and mesothorax. Fine setae in distinct submedial and marginal series; setae not detected on head and thorax. Antennae each with two long setae. Spiracles without pores.

**Figure 7.**
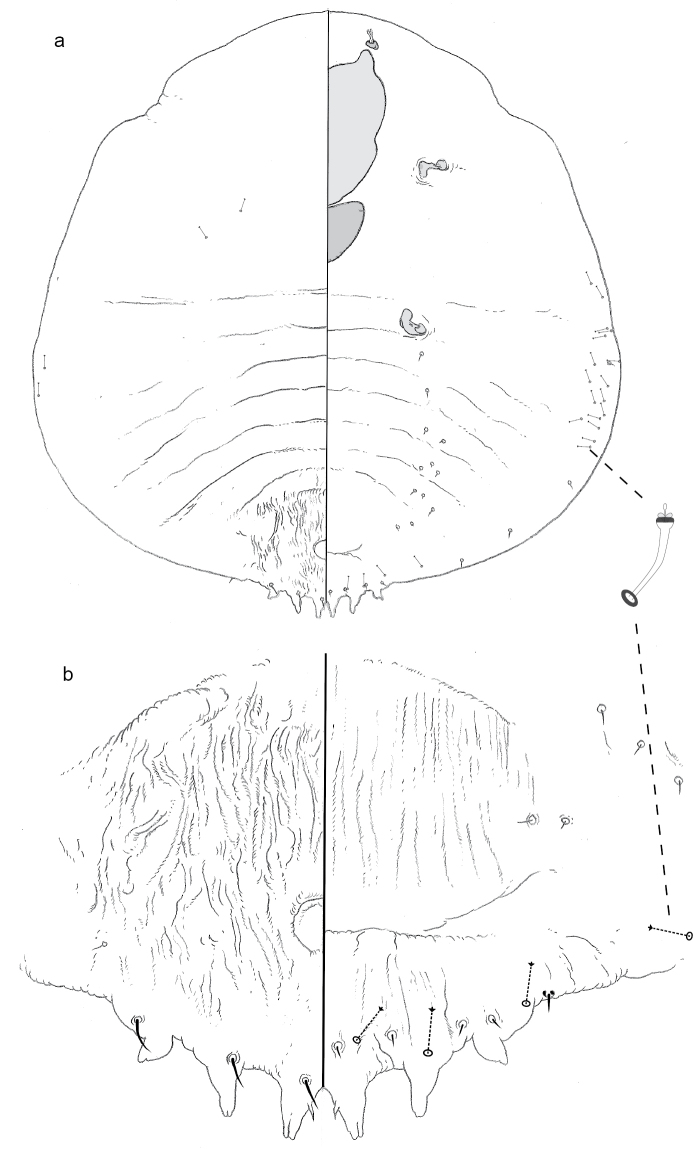
**a** Adult female of *Greeniellacasuarinae* sp. n. with **b** enlargement of pygidium.

#### Comments.

Before this work, the genus *Greeniella*Cockerell (1897) contained 14 valid species, all but two occurring in southern Asia. The exceptions are two Australian species, *G.capitata* Brimblecombe and *G.ornata* Brimblecombe. The defining feature for the group is that the pupillarial adult females have unusual pygidia, with caudal projections in addition to or instead of the normal lobes and plates. Nevertheless, the taxonomy of pupillarial armored scale insect species undoubtedly needs sorting, and the boundaries between the genera *Greeniella*, *Eugreeniella*, and *Aonidia* are blurry (B. B. Normark pers. comm.). The adult female of *G.casuarinae* is unique in having six short, sub-triangular, bifid caudal projections.

#### Etymology.

The species epithet is taken from the genus name of the host.

### Greeniella dacrydiae sp. n.

Taxon classificationAnimaliaHemipteraDiaspididae

http://zoobank.org/50D7573E-59B7-4901-8F77-EE3EB25290AD

Figures 8 , 9

#### Material examined.

*Holotype*: New Caledonia: 1 adult female (0.49 mm long, 0.38 mm wide): *ex Dacrydium auricarioides* [sic; the correct spelling of the epithet is *araucarioides*], 5 miles north of Yaté Dam Lake, 5.ix.1963, SW Brown, SWB accession 275 (USNM). *Paratype*: New Caledonia: 1 second-instar exuviae: on same slide as holotype, SWB accession 275 (USNM).

#### Description.

**Adult female, n = 1.** Pupillarial. Body of holotype 0.49 mm long, broadest at meta-thorax and anterior abdominal segments (0.38 mm); body outline ovoid, with slight constriction at head.

*Pygidium* truncate, without dorsal macroducts, or typical lobes and plates; with ~20 short papilliform projections. Anus circular, in anterior half of pygidium. Venter with vulva in posterior half, well behind anus. Perivulvar pores absent. Microducts scattered along posterior margin and submedial areas.

*Pre-pygidial segments.* Dorsum with few setae along margin. Venter with microducts scattered across abdominal segments, in clusters around each spiracle. Fine setae in distinct submedial and marginal series. Antennae each with one long setae. Spiracles without pores.

**Second-instar female.***Pygidium* with three lobes on each side; L1 longer than wide, parallel-sided, apex oblique; L2 and L3 sub-triangular, with oblique caudal edge, medial margin much longer than lateral margin. Two simple gland spines in each interlobal space, and lateral of L3. Marginal two-barred macroducts with thick sclerotization around orifice; one between L1 and L2, L2 and L3, and laterad of L3; longer slenderer macroduct arising from base of each marginal gland spine. Anus circular, near middle of pygidium.

**Figure 8.**
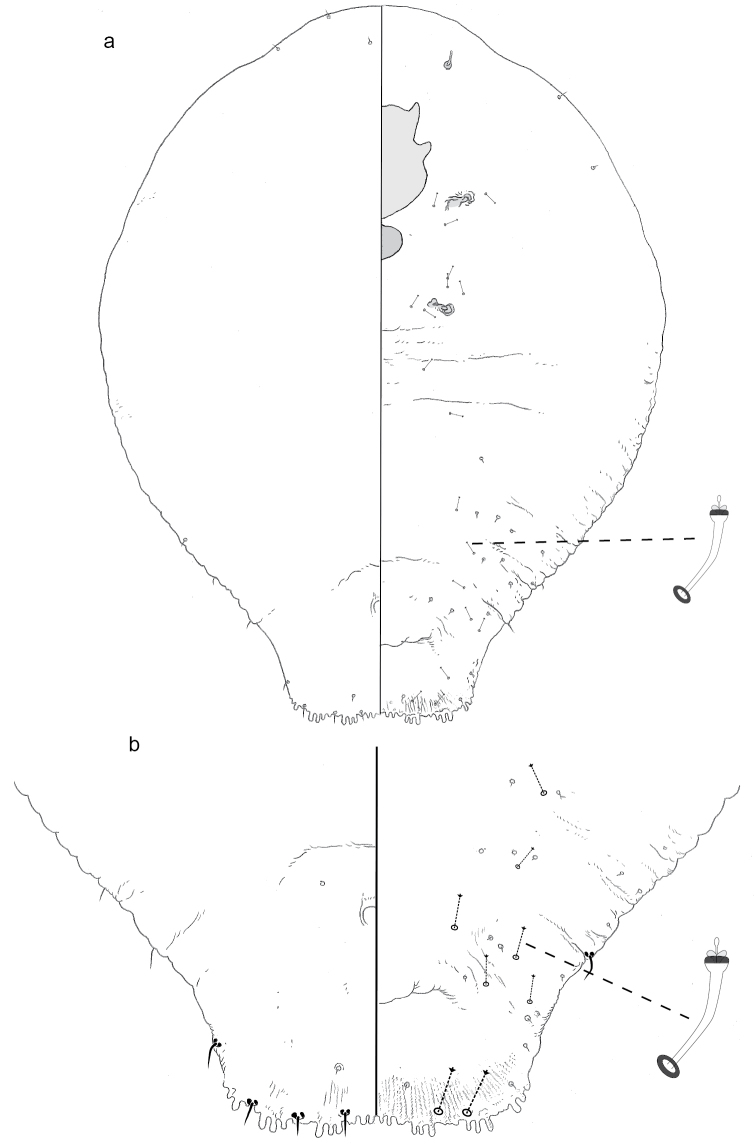
**a** Adult female of *Greenielladacrydiae* sp. n. with **b** enlargement of pygidium.

**Figure 9.**
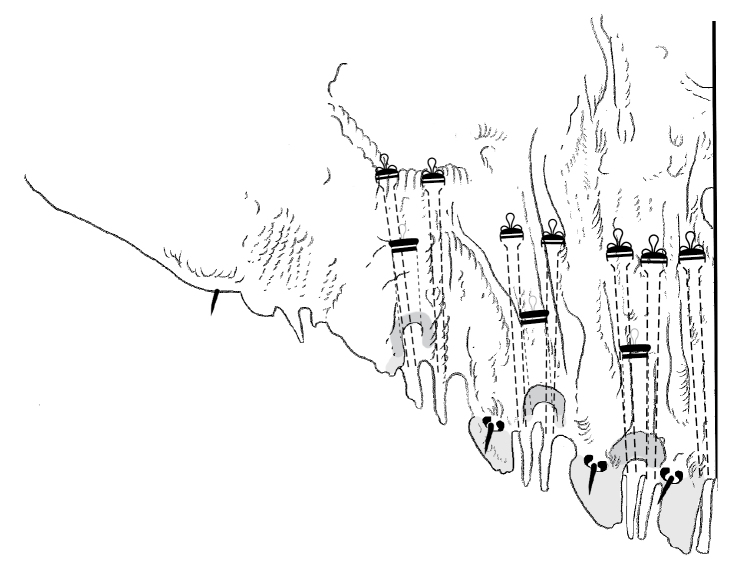
Pygidium of 2^nd^ instar of *Greenielladacrydiae* sp. n.

#### Comments.

The adult female of *G.dacrydiae* can be easily distinguished from those of other described species by the ~20 papilliform projects along the truncate posterior margin of the pygidium. See comments under *G.casuarinae* for further discussion of genus assignment.

### Lepidosaphes monticola sp. n.

Taxon classificationAnimaliaHemipteraDiaspididae

http://zoobank.org/1D189785-917C-4E7D-850E-908367B85A3E

Figure 10

#### Material examined.

*Holotype*: New Caledonia: 1 adult female (1.75 mm long, 0.75 mm wide): *ex Podocarpus ?logifolatus*, Mt. Koghis, 900 m, 12.x.1978, PN Johnson, BM 19 2 (NHMUK). *Paratypes*: New Caledonia: 1 adult female: same data as holotype; 2 adult females: *ex* undetermined host, Mt. Mou (near Sanitarium), 19.viii.1963, SW Brown, SWB accession 254 (NHMUK, USNM); 1 adult female: *ex Podocarpus longifolatus*, Mt. D’Or, 900 m, 31.x.1978, J. S. Dugdale, 78-3266 (NHMUK). *Other material*: New Caledonia: 1 teneral adult female: same data as holotype but with accession BM 19 17.

#### Description.

**Adult female, n = 4.** Presumed to secrete scale cover. Body 1.25–1.88 mm long, broadest near anterior spiracle (0.5–0.83 mm); outline roughly fusiform, head and prothorax fused into circular prosoma, margin incised between pro- and mesothorax, margins of posterior pre-pygidial abdominal segments strongly convex.

*Pygidium* with three lobes on each side, L2 and L3 bilobed, each with lateral marginal slightly divergent near base, L1 and sublobes of L2 each with pair of convergent paraphyses. A pair of bifid gland spines between L1s, a pair of simple gland spines between L1 and L2, another pair between L2 and L3. Dorsum of pygidium weakly sclerotic, with longitudinal striations. Anus small, 10 μm in diameter, in middle of pygidium. Margin of pygidium with five large two-barred macroducts (~25 μm long, 10–13 μm wide at distal end) with elongate, oblique orifices: one on pore prominence between L1 and L2, one between L2 and L3, one just anterior to first sublobe of L3, two anterolateral of L3. Other dorsal macroducts (away from margin) as long as marginal ducts but less wide at distal end, ~7 μm, scattered along submargin and arranged in longitudinal line from L3 to anterior of anus. Venter of pygidium with vulva in anterior half of pygidium. Perivulvar pores quinquelocular, 5–9 µm in diameter, in five groups: 8–11 pores in posterior group, 11–12 in anterolateral group, 3–5 in medial group.

*Prepygidial segments* Dorsum with fine, hair-like setae, sparse along margin, few on medial areas of head and thorax. Each abdominal segment with submedial and submarginal group of macroducts. In holotype, submedial group in transverse line, submarginal group divided into anterior cluster and posterior transverse line. Some other specimens with fewer ducts and without differentiated subdivisions of submarginal group. Each posterior abdominal segment with cluster of macroducts associated with 1–3 gland spines. Antennae each with two long setae. Anterior spiracle with cluster of 3–10 trilocular pores. Posterior spiracles without pores. Some specimens with distinct clusters of microducts on head or prothorax.

**Figure 10.**
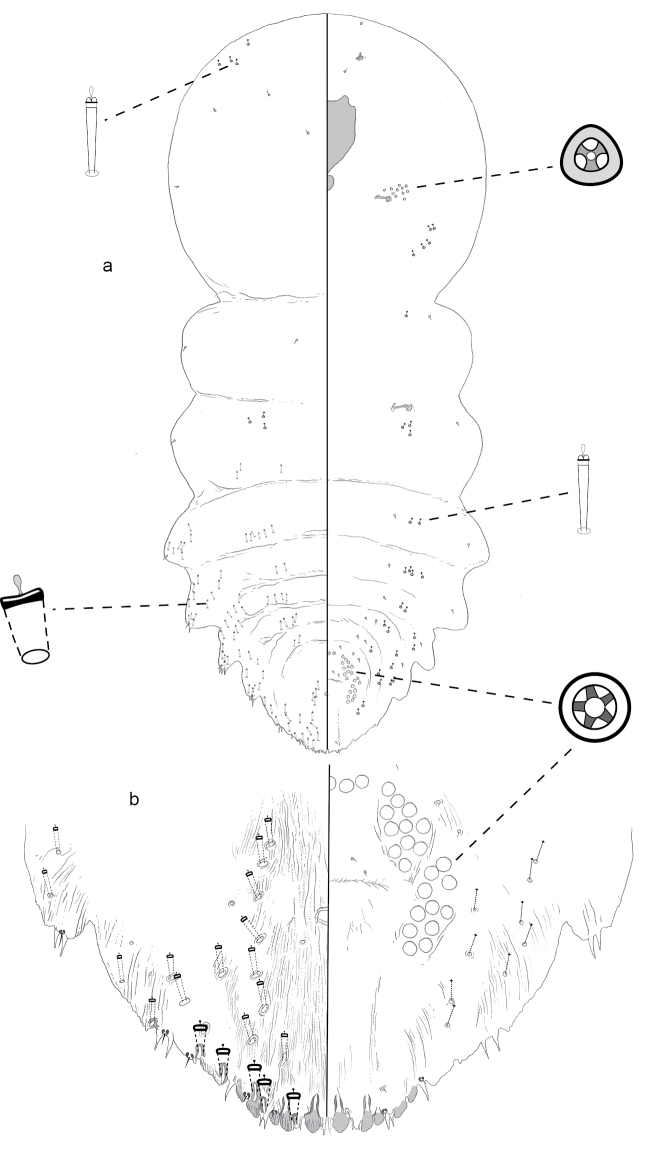
**a** Adult female of *Lepidosaphesmonticola* sp. n. with **b** enlargement of pygidium.

#### Comments.

Shimmer (1868) erected the genus *Lepidosaphes* by monotypy for the species *Mytilococcuscommunis* Amerling (now *Lepidosaphesulmi*). There are 167 species currently recognized in the genus (García Morales et al. 2016). Takagi (1970) gives a thorough diagnosis: adult females tend to have (1) an elongate or fusiform body shape, the margins of anterior abdominal segments convex; (2) two pairs of well-developed pygidial lobes, and in some species an additional 1–2 pairs of rudimentary lobes; (3) median pygidial lobes separate and symmetrical; (4) L2 bilobate; (5) if basal scleroses present on pygidium, they are slender and extend from the basal edges of lobes; (6) gland spines present between medial lobes as well as in the space between L1 and L2; (7) large marginal macroducts with oblong orifice and sclerotic rim, usually six on each side of pygidium, stereotypically with one near junction of abdominal segments VIII and VII, two near junction of segments VII and VI, and three anterior to segment VI; (8) antenna usually with two or more long setae; (9) anus near anterior edge of pygidium; (10) perivulvar pores in five groups; and (11) pores near anterior spiracles. For the most part, this diagnosis works for the adult female of *L.monticola*, although it does have some less common character states, for example L3 is relatively well developed, and the anus is farther posterior of the anterior pygidial edge (opposite the posterior group of perivular pores) than it is in most species (at the base of the pygidium or almost opposite the center of the frame formed by the perivulvar pores). The most striking difference is the circular prosoma, which, to the naked eye, makes the body of the *L.monticola* female appear unlike other species of *Lepidosaphes*. This body shape is typical of many species of *Aulacaspis* Cockerell, but the pygidium of *Aulacaspis* species is quite different (Takagi 1999). Two Neotropical species of *Pseudoparlatoria* Cockerell have a body shape and pygidium similar to that of *L.monticola* (Ferris 1941; Wolff 2001; Wolff and Claps 2008). Nevertheless, species of *Pseudoparlatoria* invariably lack pores near the anterior spiracles of the adult female. In a forthcoming paper, B. B. Normark will show that in comparison to body shape, the presence or absence of pores near anterior spiracles is more phylogenetically conservative. Hence, we describe *L.monticola* as an odd-bodied species of *Lepidosaphes*, rather than an odd-pored species of *Pseudoparlatoria*.

*Lepidosaphesmonticola* belongs to a group of Pacific species that share a row of microducts on segment VII, forwards from the second lobes. In the other Pacific species this group only extends anteriorly to each side of the anus, but in *L.monticola* this group extends well anterior to the anus. *Lepidosaphesmonticola* is similar to *L.carolinensis* Beardsley, described from The Federated States of Micronesia, *L.esakii* Takahashi, known from The Federated States of Micronesia and The Republic of Kiribati, and to *L.karkarica* Williams & Watson, described from Papua New Guinea. *Lepidosaphescarolinensis* has sclerotized spines on the lateral margins of the anterior abdominal segments, and *L.karkara* possesses well-developed lateral tubercles in these positions but *L.monticola* lacks these structures.

Note that we have excluded from the type series a teneral adult female specimen from the type locality, Mt. Koghis. This specimen differed from the others in having a distinct patch of short microducts along the submargin of the prosoma, lateral of the clypeolabral shield. The adult females from Mt. Mou also have clusters of microducts on the margin of the head, although not as many as the teneral Mt. Koghis female, and in a different location (the anterior margin). The teneral Mt. Koghis female also has smaller perivulvar pores than those found in the other specimens examined (5 versus 9 µm in diameter).

#### Etymology.

The species name refers to its mountainous habitat and is a noun in apposition.

### Leptaspis gen. n.

Taxon classificationAnimaliaHemipteraDiaspididae

http://zoobank.org/2D39CBD6-EA5C-4742-A3EB-E08F8E45DCD2

#### Type species.

*Leptaspispege* sp. n. by monotypy and original designation.

#### Diagnosis.

A pupillarial genus. Adult female with body outline elongate, nearly four times as long as wide. Pygidium without lobes, plates or macroducts. Perivulvar and perispiracular pores absent. Gland tubercles in marginal series. Eye a subcircular disk on dorsal submargin lateral of antenna. Antenna with one long fleshy seta. Second-instar female with three pairs of pygidial lobes, fimbriate plates, and slender one-barred macroducts. Anus at anterior end of medial furrow delimited by sclerotic carinae.

#### Comments.

As has been done many times before, we erect a new monotypic genus for a pupillarial species with no obvious taxonomic affinity. In this case, the adult female has the unusual combination of gland tubercles and anterior spiracles without pores. Pupillarial life histories have evolved repeatedly in armored scale insects (Andersen et al. 2010) and have spurred extensively parallel evolution of a variety of morphological reductions. Some features of the new genus are similar to those of *Neoleucaspis* Green described from India, but the latter genus possesses a series of lobe-like structures around the pygidial margin and lacks gland tubercles. Moreover, the lobes of the second-instar of *Neoleucaspis* are pointed whereas the lobes of the new genus are rounded. Because the genus is monotypic, the description of the type species also describes the genus.

### Leptaspis pege sp. n.

Taxon classificationAnimaliaHemipteraDiaspididae

http://zoobank.org/645176E4-341C-4422-8D21-EB689F1380F0

Figure 11a, b, c

#### Material examined.

*Holotype*: New Caledonia: 1 adult female (1.46 mm long, 0.38 mm wide): *ex* sedge, Mont D’Or, roadside fountain, 24.viii.1963, SW Brown, SWB accession 257 (USNM). *Paratype*: New Caledonia: exuviae of 1 second-instar: on same slide as holotype, SWB accession 257 (USNM).

#### Description.

**Adult female, n =1.** Pupillarial. Body of holotype 1.46 mm long, broadest at meta-thorax (0.38 mm); body outline elongate.

*Pygidium* without lobes or plates, on each side with ~10 microducts along margin. Anus circular, in posterior third of pygidium. Venter with vulva in posterior half, slightly anterior of anus. Perivulvar pores absent.

*Pre-pygidial segments.* Eye well developed. Dorsum with few setae along margin and medial areas. Venter with gland tubercles, in linear submarginal series along abdominal segments, a few on margin of metathorax, and extending from anterior spiracle to antenna. Fine setae in distinct submedial and marginal series. Antennae each with one long setae. Spiracles without pores.

**Second-instar female.***Pygidium* with three lobes on each side; each lobe with rounded apex; L1 much smaller than L2 and L3. Medial side of base of L2 and L3 confluent with sclerotized rim around orifice of marginal microduct. Microducts also scattered along submargin. Two fimbriate plates between medial lobes, two between L1 and L2, 3 between L2 and L3, 2 laterad of L3. Anus elongate, at anterior end of medial furrow extending from base of medial lobes.

**Figure 11.**
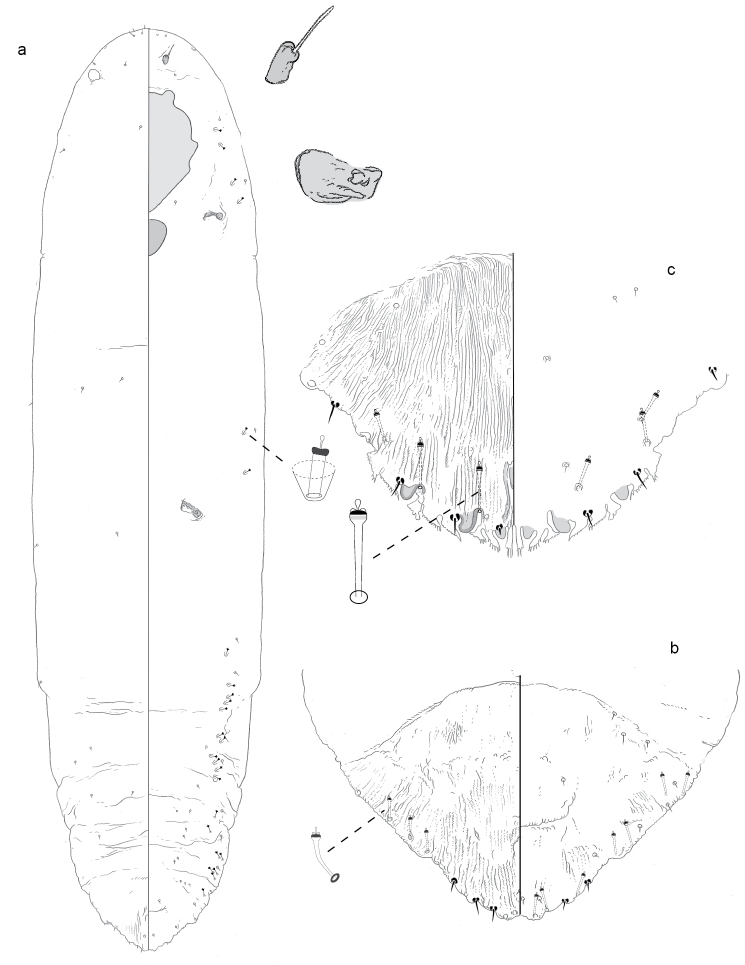
**a** Adult female of *Leptaspispege* gen. et sp. n. with enlargements of pygidium of **b** adult female and (c) 2^nd^-instar.

#### Etymology.

The genus name is based on the Greek word *leptos* meaning thin, referring to the shape of the body, combined with *aspis*, the Greek world for shield. The species name is taken from the Greek *pege*, for fountain, in reference to the one located near the type locality. It is a feminine noun, used here in apposition.

### Leucaspis montikoghis sp. n.

Taxon classificationAnimaliaHemipteraDiaspididae

http://zoobank.org/4B28BBB5-529C-4A9D-8C89-1BDBD7A5A0DB

Figure 12

#### Material examined.

*Holotype*: New Caledonia: 1 adult female (2.06 mm long, 0.54 mm wide): *ex Podocarpus* sp., Mt. Kohgis, 12.x.1978, PN Johnson, BM 19 17 (NHMUK). *Paratypes*: New Caledonia: 2 adult females, 6 puparia (2 of which contain embryos), and 1 first-instar nymph on 9 slides: same data as holotype, BM 19 17 (NHMUK, USNM). Note that one slide further specifies that the elevation was 900 m, and that a possible species assignment for the host was *P.longifollatus*.

#### Description.

**Adult female, n = 3.** Pupillarial. Body 0.94–2.06 mm long, 0.51–0.54 mm wide; outline elongate, margins of head and pygidium rounded.

*Pygidium* with two lobes on each side, each longer than wide, lanceolate (i.e., distal half tapering to pointed apex), base slightly overlaying venter of pygidium. Plates spiniform, slightly longer than lobes, two between L1s, two between L1 and L2, ~8 anterolateral of L2. Dorsum with sclerotic area containing anus, plus two smaller patches posterior to anus, medial patch confluent with that around anus in some specimens; anus ~20 μm long and ~15 μm wide. Few small ducts scattered along posterior margin, each ~5 µm long. Venter of pygidium with perivulvar pores in five distinct groups, each side of the body with a lateral group of ~20 pores, and an anterolateral group of ~25 pores, anteromedial group with 10–20 pores.

*Prepygidial segments* Dorsum with few fine, hair-like setae. On venter, four groups of pre-pygidial pores, one group of 12–20 pores on the submargin of each of abdominal segments IV-VI, plus one group of 2–6 pores on submedian area of segment VI. Longitudinal band of 65–85 gland tubercles running from anterior to spiracle to posterior of labium. Antennae each with five long setae, two short ones evidenced by sockets. Anterior spiracles each with cluster of 24–28 quinquelocular pores. Posterior spiracle without pores.

**Figure 12.**
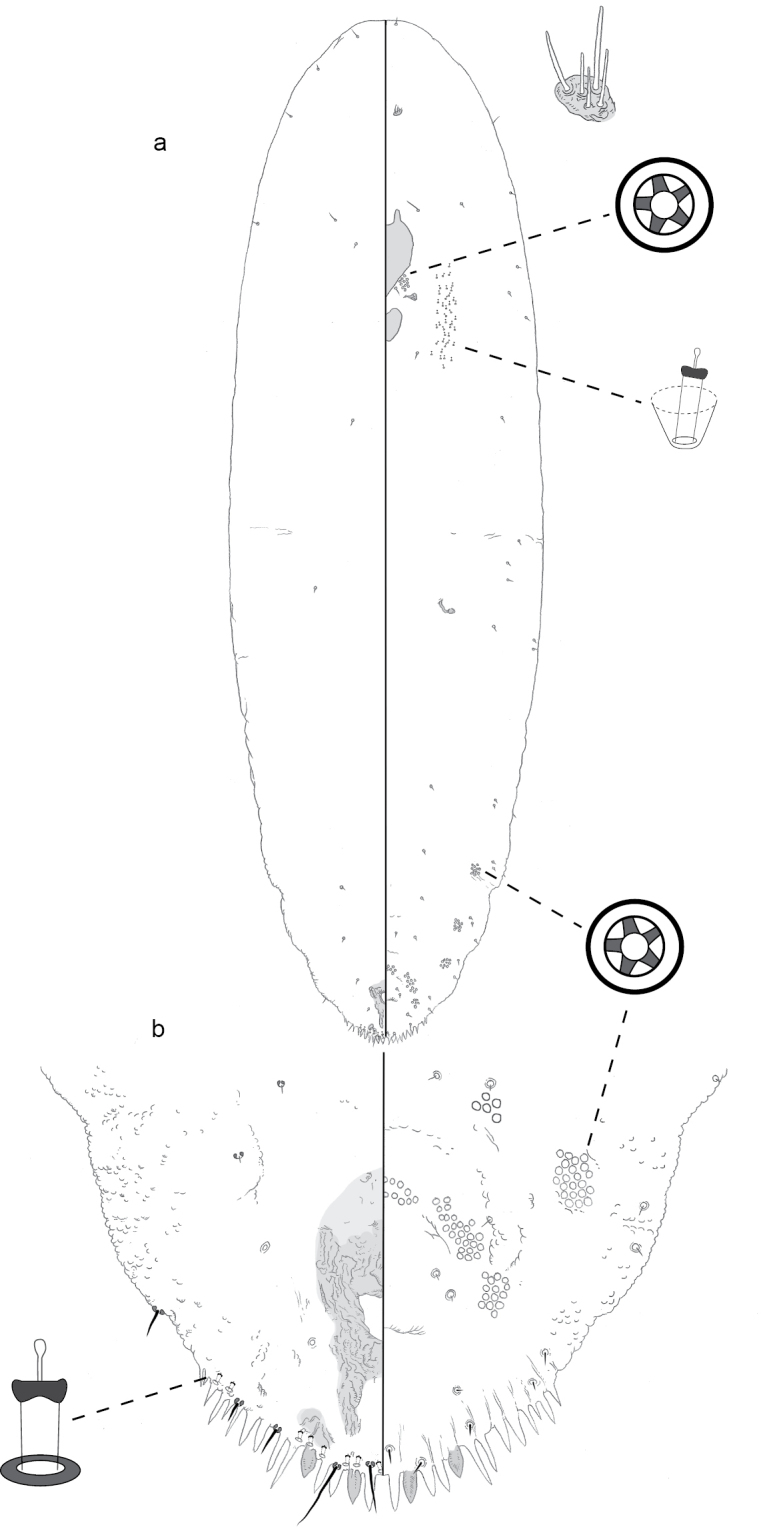
**a** Adult female of *Leucaspismontikoghis* sp. n. with enlargement of **b** pygidium.

#### Comments.

The genus *Leucaspis* Signoret was erected for the type species *Aspidiotuspini* Hartig. There are 34 nominal species of *Leucaspis* (García Morales et al. 2016). Nineteen of these occur in New Zealand (~2000 km south of New Caledonia), 16 of which are endemic, and one of which is only known from New Zealand and Australia (which is home to only this one species of *Leucaspis*). The true species diversity of New Zealand’s *Leucaspis* is apt to be much greater (Henderson 2011; personal observations of material in the USNM). The species *L.bugnicourti* Cohic is endemic to New Caledonia, but would appear to be more closely related to species outside of *Leucaspis*, for example *Fijifiorinia* spp. (Williams and Watson 1988). Takagi (1969) gives us a meticulous diagnosis of the genus. We paraphrase it here. The adult female can be recognized by (1) being pupillarial, and by having (2) an elongate body outline; (3) small sclerotic patches on dorsum of pygidium; (4) each side of the pygidium with 1–4 lobes, with medial lobes well separated; (5) plates absent, spiniform or fimbriate; (6) gland tubercles on venter of thorax; (7) dorsal ducts absent or present only along the pygidial margin, when present short, with sclerotized oral rim; (8) antenna with 2–6 fleshy setae; (9) anterior spiracle with cluster of disc pores adjacent; (10) anus in anterior third of the pygidium; (11) perivulvar disc pores in five groups; (12) many species with pre-pygidial pores. The new species, *L.montikoghis*, can be recognized by having two pairs of broad lanceolate pygidial lobes, spiniform plates, and many pores (~20) in the posterior pre-pygidial group. The latter trait is especially distinctive; there are about twice as many pores in this group as in any described species.

Brittin (1937) provides a synthetic treatment of the *Leucaspis* species occurring in New Zealand that he was aware of at that time (13 in all). We recommend this, along with Takagi’s diagnosis, as good resources to start gaining familiarity with this group. de Boer and Valentine (1977) provide an excellent re-description of *L.gigas*, along with four similar species, one of which was new at that time. Henderson et al. (2010) described an additional two New Zealand species.

#### Etymology.

The species epithet is taken from the type locality and is a noun in apposition.

### Melanaspis nothofagi sp. n.

Taxon classificationAnimaliaHemipteraDiaspididae

http://zoobank.org/7AA6F01A-37FD-484B-9523-928275B99768

Figure 13

#### Material examined.

*Holotype*: New Caledonia: 1 adult female (0.79 mm long, 0.74 mm wide): *ex Nothofagus aequilateralis*, Pic du Pin, 6.x.1978, JS Dugdale, BM 19 7 (NHMUK).

#### Description.

**Adult female, n = 1.** Presumed to secrete scale cover. Body 0.79 mm long, 0.74 mm wide; outline circular, margin of posterior abdominal segments sinusoidal, i.e., appearing lobed, posterior part of each lobe bearing sclerotic tooth, tip pointed or rounded.

*Pygidium* with four lobes on each side of body, each roughly rectangular in shape, with apex notched or emarginate. L1 longer than wide; L2, L3, and L4 wider than long. One linear paraphysis between L1s, two paraphyses in every other interlobal space, lateral one in each pair near medial base of lobe delimiting lateral edge of interlobal space, this lateral paraphysis much shorter than medial one in same interlobal space. One simple plate with blunt apex between L1 and L2, and another between L2 and L3 (these are difficult to make out on holotype). Dorsum of pygidium with subtriangular area of smooth sclerotic cuticle, lateral edges converging to space between L2 and L3, two smaller elongate, oblique sclerites lateral of this sclerotic area, separated by furrows of membranous cuticle, the medial one near L3 and the lateral one anterior of L4. One-barred macroducts near L2, in and near furrows between sclerites, and along margin, decreasing in length anterolaterally. Anus small and compressed lateromedially (~5 μm wide and 15 μm long) in the posterior half of pygidium. Venter of pygidium with vulva in anterior half. One cluster of 8–10 quinquelocular perivulvar pores anterolaterally of each side of vulva.

*Pre-pygidial segments.* Dorsum with fine, hair-like setae, especially dense along margin, decreasing in length mesally. one-barred macroducts much shorter than those on posterior pygidial segments (~13 μm long scattered along margin of abdomen. Microducts scattered along submargin. On venter, microducts scattered along submargin of anterior abdominal segments, plus a few near posterior spiracle. Small setae in distinct longitudinal submedial and submarginal lines across abdominal segments, a few additional setae between these lines. Antenna with one long seta, one short seta evident from socket. No pores near spiracles.

**Figure 13.**
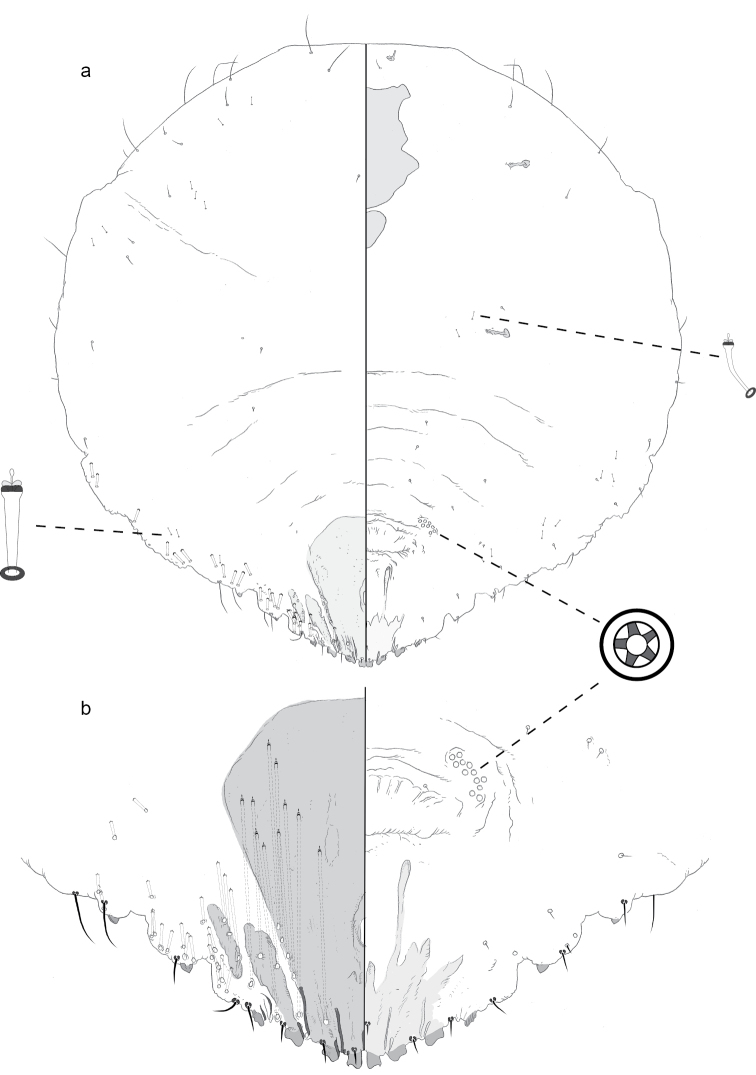
**a** Adult female of *Melanaspisnothofagi* sp. n. with enlargement of **b** pygidium.

#### Comments.

With 63 described species, *Melanaspis* Cockerell is one of the more diverse genera of armored scale insects (García Morales et al. 2016). The type species is *Aspidiotusobscurus* Comstock. They occur world-wide, but more than half of the species (35) can be found in the Nearctic Region. Only *Melanaspisbromiliae* has been recorded from the South Pacific (in Guam). If we liberally define the Australasian biogeographic zone in a way that extends as far north as the Bonin Islands, we can also find *Melanaspismarlatti* (Parrott). Both of those species feed on monocots. If we cast our net even further afield, to include the Indian Ocean, we find five species recorded from Madagascar: *M.artemisiae* Mamet, *M.casuarinae* Mamet, *M.madagascariensis* Mamet, *M.philippiae* Mamet, and *M.sansevii* Mamet.

Following the generic diagnosis of Dietz and Davidson (1986), the adult female of *Melanaspis* species have (1) a circular body outline; (2) four lobes on each side of pygidium; (3) paraphyses arising from interlobal spaces, and in some species the bases of lobes or the margin up to a short distance anterior to L4; (4) dorsum of pygidium with a large medial sclerotic area, and a smaller sclerotic strap extending anterolaterally from L3 and L4; (5) interlobal areas with small plates, each with simple or slightly fringed apex. With the addition of this new species, *M.nothofagi*, no change to this diagnosis is necessary. The adult female of *M.nothofagi*, can be recognized by having (1) one group perivulvar pores on each side; (2) sclerotic teeth on marginal protrusions of posterior pre-pygidial abdominal segments; (4) a pair of linear apophyses in each interlobal area; (5) no other apophyses; (6) a sclerotic tooth on posterior of marginal lobes of posterior pre-pygidial segments.

#### Etymology.

The epithet is taken from the genus name of the host plant genus *Nothofagus*.

### Neomorgania nothofagi sp. n.

Taxon classificationAnimaliaHemipteraDiaspididae

http://zoobank.org/60F7F46C-7473-49D1-9D35-B63B8510CFEE

Figure 14

#### Material examined.

*Holotype*: New Caledonia: 1 adult female (1.08 mm long, 0.76 mm wide): *ex Nothofagus codonandra*, Riviera Bleue, 10.x.1978, JS Dugdale, BM 19 11 (NHMUK). *Paratypes*: New Caledonia: 5 adult females on 5 slides: same data as holotype, BM 19 11 (NHMUK, USNM); 6 adult females and 1 second-instar nymph on 7 slides: *ex Nothofagus baumanii*, Mt. Mou, 2.xi.1978, PN Johnson, BM 19 5, BM 19 20 (NHMUK, USNM, MNHN).

#### Description.

**Adult female, n = 12.** Presumed to secrete scale cover. Body 1.07–1.56 mm long, broadest near posterior end of fused head and prothorax (0.76–1.17 um); outline roughly turbinate (head and thorax broad, abdomen tapering caudally), deeply incised between thoracic segments and between posterior pre-pygidial abdominal segments.

*Pygidium* only with one lobe, L1, on each side of body, triangular in shape, with medial edge in close proximity to midline and parallel to it, apex oblique, extending to body margin. Dorsum of pygidium with subtriangular sclerotic area of smooth cuticle, lateral edges converging to posterior margin lateral of L1, with narrow, bifurcate groove in sclerotic area lateral of L1, one smaller oblique sclerite lateral of main sclerite, separated from it by membranous furrow. Anus mediolaterally compressed, ~12 µm long, 4 µm wide, in posterior third of pygidium, at anterior end of triangular, medial groove in sclerite. Two simple plates, each with blunt apex, laterad of L1 on each side of body, at base of membranous furrows. One-barred macroducts in base of bifurcate groove lateral of L1, in furrow between central sclerite and lateral sclerite, in line along submargin, decreasing in size anterolaterally. Venter of pygidium with vulva in anterior half. One cluster of perivulvar pores (30–41) on each side of body, anterolaterally of vulva.

*Prepygidial segments* Dorsum with fine, hair-like setae, scattered along margin, decreasing in length mesally, one in each side of submargin and one in the submedial area of meso- and meta-thorax, only submedial seta present on prothorax. Cluster of short macroducts (~5 μm) on submargins of fused head + prothorax. Microducts in submedial clusters on each thoracic segment and anterior abdominal segments. On venter, microducts scattered along submargin of anterior abdominal segments, a few near anterior spiracle, surrounding and mixed in with cluster of disc pores. Small setae in distinct longitudinal submedial and submarginal lines across abdominal segments, a few additional setae between these lines. Antennae each with one long seta, one small seta evidenced by second socket. Large cluster of 40–55 quinquelocular pores medial of anterior spiracles. Posterior spiracles without pores.

**Figure 14.**
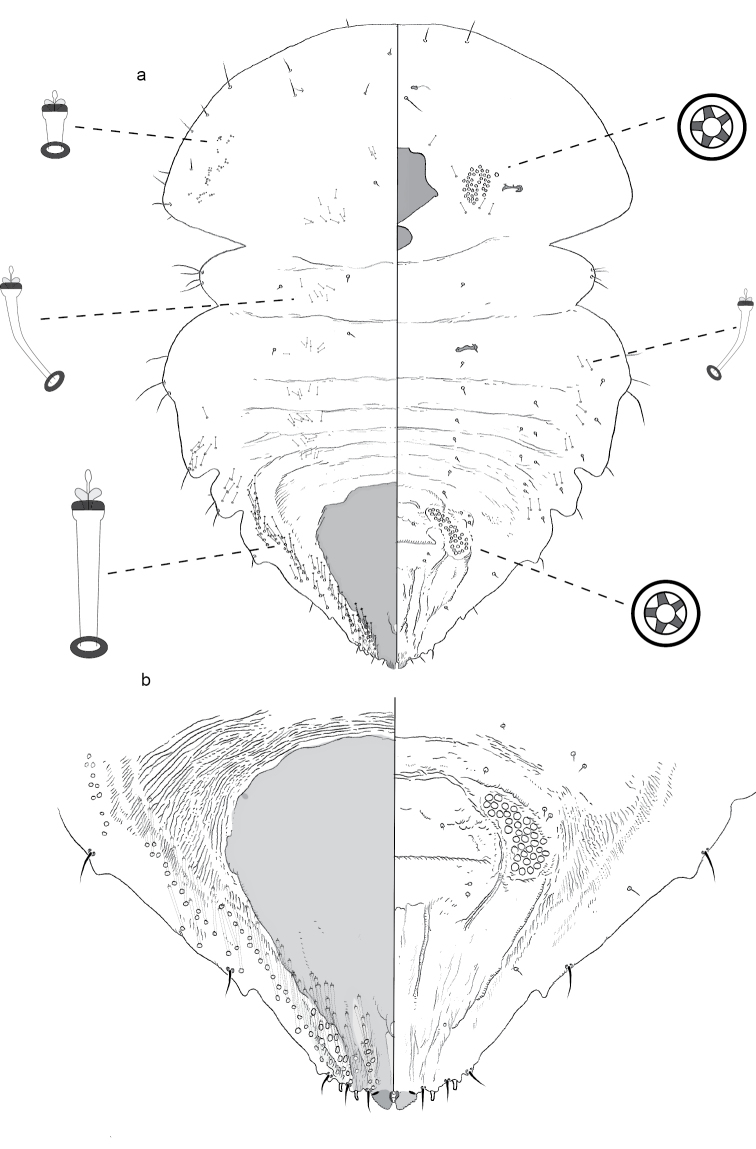
**a** Adult female of *Neomorganianothofagi* sp. n. with enlargement of **b** pygidium.

#### Comments.

The genus *Neomorgania* was erected by MacGillivray (1921) for the three species *Aspidiotusjunctiloba* Marlatt, *A.acaciae* Morgan, and *A.eucalypti* Maskell. Brimblecombe (1954) considered these to be one and the same, and synonomized the names under *Neomarganiaeucalypti*, which Ferris (1941) had designated as the type species of the genus. Following Brimblecombe (1954), the following characters are diagnostic of *Neomorgania*: (1) margin of thorax with pronounced incision; (2) pygidium with only one pair of lobes, L1s, which are adpressed but not fused beyond base; (3) basal scleroses absent; (4) paraphyses present: in *N.eucalypti* these are distinct only at the lateral edge of the medial lobes, and are slightly longer than the lobes themselves; (5) no more than a single small plate lateral of the medial lobe; (6) one-barred macroducts smaller than the average across armored scale species; (7) anus small, at anterior end of triangular groove that terminates between the medial lobes; (8) margin of pygidium crenulate near medial lobes; (9) perivulvar pores absent. With a few of exceptions, the adult female of the new species, *N.nothofagi*, fits each of these definitions. The exceptions are that it has one group of perivulvar pores on each side of the body, it has two simple plates on each side of the pygidium, and the paraphyses are shorter than the medial lobes. *N.nothofagi* can also be distinguished by having the body outline incised between the meso- and metathorax, in addition to between the pro- and mesothorax. Furthermore, it has a large cluster of quinquelocular pores mesal of each anterior spiracle; pores are absent from this location in Brimblecombe’s illustration of *N.eucalypti*, and he does not mention them in his diagnosis.

#### Etymology.

We follow the practice of previous taxonomists, and take the species epithet of a *Neomorgania* species from its host plant, in this case *Nothofagus*.

### Pseudaonidia dugdali sp. n.

Taxon classificationAnimaliaHemipteraDiaspididae

http://zoobank.org/F94537B5-1798-430D-97BD-6E2ED6725BF5

Figure 15

#### Material examined.

*Holotype*: New Caledonia: 1 adult female (1.64 mm long, 1.32 mm wide): *ex Nothofagus aequilateralis*, Pic du Pin, 6.x.1978, JS Dugdale, BM 19 7 (NHMUK). *Paratypes*: New Caledonia: 7 adult females (5 damaged by fungus) and 1 second-instar male on 8 slides: same data as holotype, BM 19 7, 16 (NHMUK, USNM, MNHN). *Other material*: New Caledonia: 1 adult female: same data as holotype, BM 19 16; 1 second-instar female: *ex N. aequilateralia*, Ridge of Pic du Amua, 26.x.1978, JS Dugdale, BM 19 24 (NHMUK).

#### Description.

**Adult female, n = 8.** Presumed to secrete scale cover. Body 1.22–1.53 mm long, broadest near posterior end of prosoma, that is, fused head and prothorax (0.82–1.01 mm); body outline roughly turbinate (head and thorax broad, abdomen tapering caudally), incised between pro- and mesothorax, each pre-pygidial abdominal segment with membranous tooth on margin, each tooth with seta at base.

*Pygidium* with three well-developed lobes on each side, each roughly rectangular in shape, with distinct longitudinal striations, notch on lateral corner of apex, small paraphysis extending longitudinally from near medial edge. Each lobe extending out from body margin approximately parallel to longitudinal body axis. A small sclerotic tooth anterolateral of L3 may represent L4. Two fimbriate plates between medial lobes (L1), two between L1 and L2, 2–3 between L2 and L3, 3 anterior of L3. Pygidial margin serrate anterior to L3. Dorsum of pygidium with large medial sclerite, subtriangular, with lateral edges converging to base of L2 on each side, texture reticulate, becoming striate posteriorly, short furrow of membranous cuticle between L1 and L2, not reaching anus. Two additional dorsal sclerites, each with striated texture, first extending anterolateral from base of L3, separated from medial sclerite by membranous furrow, second lateral of the first, with another membranous furrow between them. Anus small, lateromedially compressed (15 μm long, 10 μm wide) in posterior third of pygidium. One-barred macroducts mostly in membranous furrows between sclerites, orifice of posterior-most duct in each furrow with heavy sclerosis on one or both sides. Venter of pygidium with vulva in anterior half. One cluster of 12–18 perivulvar pores, anterolateral of vulva.

*Prepygidial segments* Dorsum with fine, hair-like setae decreasing in size posteriorly, scattered along submargin and mid line of head and prothorax, one in the submargin and one in the submedial area of meso- and meta-thorax, along with anterior abdominal segments. Microducts scattered along submargin of head and prothorax. One-barred macroducts present along margin of abdomen. On venter, microducts scattered along medial and submarginal areas of abdominal segments, a few near anterior spiracle. Antenna with one long seta, socket of second, short seta evident. Anterior spiracle with cluster of 4–6 quiquelocular pores. Posterior spiracle without pores.

**Figure 15.**
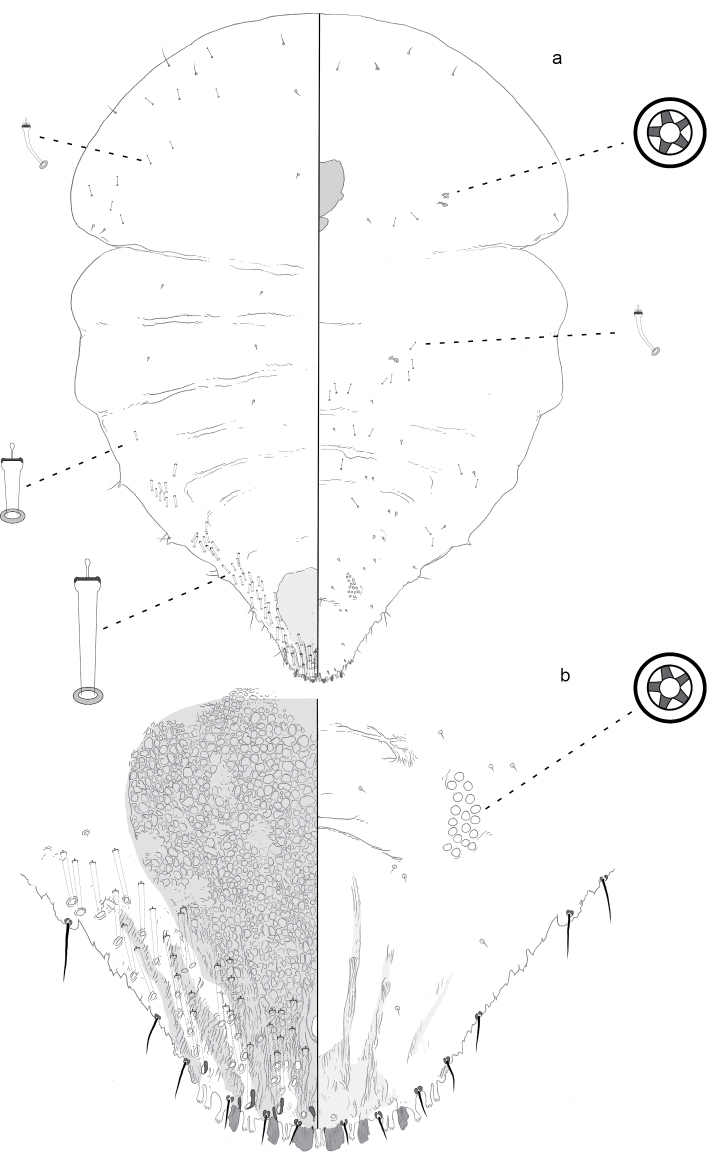
**a** Adult female of *Pseudaonidiadugdali* sp. n. with enlargement of **b** pygidium.

#### Comments.

Cockerell (1897) erected *Pseudaonidia* for the species *Aspidiotusduplex* Cockerell. Prior to this work, 20 species were recognized, five of which are presumed to be endemic to Australia (García Morales et al. 2016). Feng and Wei (2011) provide a diagnosis. Adult females of *Pseudaonida* have (1) a deep constriction of the body outline between the prothorax and mesothorax; (2) reticulations on the pygidial dorsum; (3) four pairs of pygidial lobes, each similar in shape and parallel to the longitudinal axis of the body; (4) well-developed plates; (5) paraphyses present or absent; if present, short; (6) slender, one-barred dorsal macroducts, each with a sclerotic rim around orifice; (7) submarginal macroducts in pore furrows; (8) the antenna with one long seta; (9) quinquelocular pores near each anterior spiracle; (10) anus small, in posterior third of pygidium; (11) the vulva anterior to anus; and (12) perivulvar pores present or absent. The adult female of *P.dugdaleii* fits each of these specifications. It can be distinguished from its congeners by (1) having a membranous tooth-like projection on the posterolateral corner of each prepygidial abdominal segment, (2) lacking perivulvar pores posterior to the vulva, and (3) having macroducts near the posterior margin of pygidium with heavy sclerotization along one or both sides of the orifice.

We have excluded one adult female specimen from the type series, although it was part of the same collection event (and presumably from the same host plant). We also excluded this specimen from the description above. This specimen differs from the others in several ways. Specifically, it (1) is longer, 2.14 mm, 40% longer than the next longest specimen; (2) has more quinquelocular pores near the anterior spiracles (11, whereas the others have between 4 and 6); (3) lacks distinct marginal teeth on pre-pygidial abdominal segments; and (4) has a well-developed L4. In all other respects, it looks like the other females in the same lot.

#### Etymology.

The species epithet is a patronym in honor of John S. Dugdale, who collected much of the material on which this study is based.

### Pseudaonidia yateensis sp. n.

Taxon classificationAnimaliaHemipteraDiaspididae

http://zoobank.org/B9760573-24DC-4A51-AA01-D12996E7C264

Figure 16

#### Material examined.

*Holotype*: New Caledonia: 1 adult female (0.66 mm long, 0.51 mm wide): ex *Citrus* sp., Yaté, 18.xii.2013, S Cazères, 722 7, COCHE/40/17 (NHMUK). *Paratypes*: New Caledonia: 1 damaged adult female and 1 exuviae of second-instar on same slide as holotype (NHMUK).

#### Description.

**Adult female, n = 2.** Presumed to secrete scale cover. Body 0.62–0.66 mm long, broadest near anterior end of abdomen (0.51–0.54 mm); body outline roughly ovate.

*Pygidium* with four lobes on each side, each roughly rectangular in shape, longer than wide, each extending out from body margin approximately parallel to longitudinal body axis, with notch on lateral corner of apex, medial lobes each with additional notch on medial corner. Two fimbriate plates between medial lobes (L1), three between L1 and L2, three between L2 and L3, three anterior of L3. Pygidial margin serrate anterior to L3. Dorsum of pygidium with large medial sclerotic area, subtriangular, with lateral edges converging to base of L2 on each side, texture reticulate, becoming striate posterior of anus, area of membranous cuticle between L1 and L2 expanding cephalad and extending to anus. Two additional sclerotic areas on each side of the dorsum, each with striated texture, first area may be subdivided into two sclerotic patches, extending anterolateral from base of L3, separated from medial sclerite by membranous furrow, second area lateral of the first, with another membranous furrow between them. Anus lateromedially compressed (15 µm long, 7 μm wide) in posterior third of pygidium. One-barred macroducts mostly in sclerotic areas. Venter of pygidium with vulva in anterior half. One cluster of 22–24 perivulvar pores, anterolateral of vulva, a second cluster of 13–20 pores posterolateral of vulva; on one side of body of holotype, antero- and posterolateral clusters contiguous; on paratype, one pore present anteromedial of vulva. Microducts scattered along submargin.

*Prepygidial segments* Dorsum with hair-like setae scattered along submargin of head and thorax, one smaller seta in submedial area of mesothorax, metathorax and anterior abdominal segments, one long seta on posterolateral corner of each thoracic and prepygidial abdominal segment. One-barred macroducts dense along margin of abdomen. A few microducts intermixed. On venter, microducts scattered along submarginal areas of thorax and abdomen, a few near each spiracle. Antenna with one long seta. Anterior spiracle with cluster of 12–15 quinquelocular pores. Posterior spiracle without pores.

**Figure 16.**
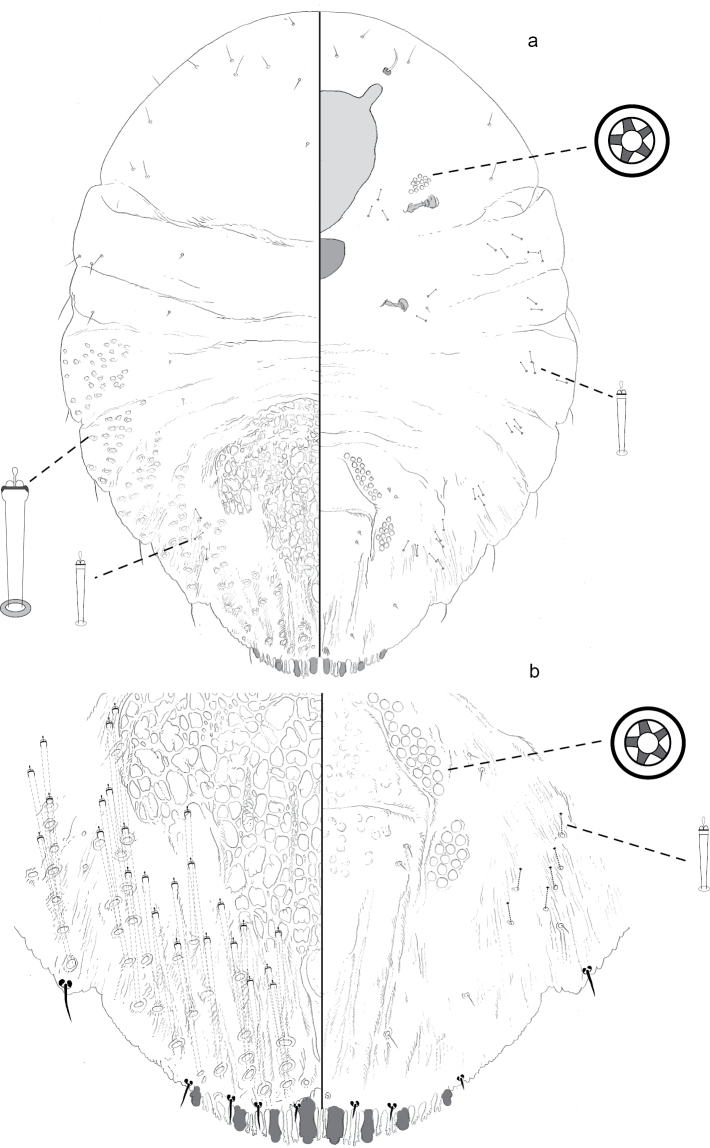
**a** Adult female of *Pseudaonidiayateensis* sp. n. with enlargement of **b** pygidium.

#### Comments.

The adult female of *P.yatensis* can be easily distinguished from that of *P.nothofagi*, by (1) having four groups of perivular pores (only two in *P.nothofagi*); (2) having more pores near the anterior spiracle; (3) having most of the dorsal macroducts arise from sclerotic areas of cuticle; (4) lacking membranous teeth on margins on abdominal segments; and (5) lacking pygidial paraphyses.

#### Etymology.

The species epithet refers to its provenance, Yaté, in the South Province of New Caledonia.

## Conclusions

We have added fourteen species to the tally of armored scale insects that are endemic to New Caledonia. Given how little the New Caledonian armored scale insect fauna has been surveyed, we have every reason to suspect that a considerable amount of the species diversity is yet to be discovered. Looking at the current catalog, we can see that it has been independently colonized by armored scale insect lineages at least 17 times. This number is the most conservative estimate; it excludes non-endemic species that may have been brought to New Caledonia by people. For example, this is likely for cosmopolitan species such as *Aspidiotusnerii* (Bouche). On the other hand, this also excludes some lineages that are apt to have crossed an ocean without our help, for example *Lindingaspisbuxtoni* (Laing), which has been recorded only from New Caledonia and western Samoa. If we take the current catalog at face value, it also suggests that trans-oceanic founder events may have been an especially important generator of new species diversity. Of 28 endemic species, 16 appear to share a most recent common ancestor with another New Caledonian endemic: the five species of *Andaspis* described by Hamilton et al. (2017), three species of *Furcaspis*, and two species each of *Agrophaspis*, *Aonidia, Greeniella*, and *Pseudaonidia*. This could simply be an indication that the current catalog only scratches the surface of the endemic species diversity. If not, and *in situ* speciation of armored scale insects is relatively rare in a place where it has been explosive in other lineages, this would pose an interesting biogeographical question.

## Supplementary Material

XML Treatment for Agrophaspis ansevatae

XML Treatment for Aonidia montikoghis

XML Treatment for Aonidia pauca

XML Treatment for Fernaldanna whita

XML Treatment for Furcaspis costulariae

XML Treatment for Greeniella casuarinae

XML Treatment for Greeniella dacrydiae

XML Treatment for Lepidosaphes monticola

XML Treatment for Leptaspis

XML Treatment for Leptaspis pege

XML Treatment for Leucaspis montikoghis

XML Treatment for Melanaspis nothofagi

XML Treatment for Neomorgania nothofagi

XML Treatment for Pseudaonidia dugdali

XML Treatment for Pseudaonidia yateensis

## References

[B1] Andersen JC Wu J Gruwell ME Gwiazdowski R Santana SE Feliciano NM Morse GE Normark BB (2010) A phylogenetic analysis of armored scale insects (Hemiptera: Diaspididae), based upon nuclear, mitochondrial, and endosymbiont gene sequences. Molecular Phylogenetics and Evolution 57: 992–1003. 10.1016/j.ympev.2010.05.002 20460159

[B2] Borchsenius NS Williams DJ (1963) A study of the types of some little-known genera of Diaspididae with descriptions of new genera (Hemiptera: Coccoidea). British Museum (Natural History) Entomological Bulletin 13: 353–394.

[B3] Brimblecombe AR (1954) Studies of Coccoidea. 2. Revision of some of the Australian Aspidiotini described by Maskell. Queensland Journal of Agricultural Science 11: 149–160.

[B4] Brimblecombe AR (1958) Studies of the Coccoidea. 7. New designations of some Australian Diaspididae. Queensland Journal of Agricultural Science 15: 59–94.

[B5] Brittin G (1937) Notes on the genus *Leucaspis*, with descriptions of thirteen New Zealand species and redescription of eight foreign species. Transactions and Proceedings of the Royal Society of New Zealand 67: 281–301.

[B6] Cockerell TDA (1897) The San Jose scale and its nearest allies. United States Department of Agriculture, Division of Entomology, Technical Series 6: 1–31.

[B7] de Boer JA Valentine EW (1977) The identity of *Leucaspisgigas* (Homoptera: Diaspididae), with descriptions of four similar species in New Zealand, New Zealand Journal of Zoology 4: 153–164

[B8] Deitz LL Davidson JA (1986) Synopsis of the armored scale genus *Melanaspis* in North America (Homoptera: Diaspididae). North Carolina State University, Technical Bulletin No. 279: 1–91.

[B9] Feng J-N Wei J-F (2011) A new species of revision of [sic] the genus *Pseudaonidia* Cockerell, 1897 (Hemiptera: Coccoidea: Diaspididae: Aspidiotinae), with description of one new species and a chicklist [sic] of the species from the Oriental Region. Transactions of the American Entomological Society 137: 173–178.

[B10] Ferris GF (1941) Atlas of the scale insects of North America. Series 3. Stanford University Press Palo Alto, California, 240 pp.

[B11] García Morales M Denno BD Miller DR Miller GL Ben-Dov Y Hardy NB (2016) ScaleNet: a literature-based model of scale insect biology and systematics. Database: The Journal of Biological Databases and Curation 2016: bav118. 10.1093/database/bav118 PMC4747323 26861659

[B12] Grandcolas P Murienne J Robillard T Desutter-Grandcolas L Jourdan H Guilbert E Deharveng L (2008) New Caledonia: a very old Darwinian island? Philosophical Transactions of the Royal Society B 363: 3309–3317. 10.1098/rstb.2008.0122 PMC2607381 18765357

[B13] Hamilton FB Williams DJ Hardy NB (2017) Five new species of *Andaspis* (Hemiptera, Coccomorpha, Diaspididae) from New Caledonia. ZooKeys 693: 17–31. 10.3897/zookeys.693.13074 PMC5672737 29133992

[B14] Henderson RC (2011) Diaspididae (Insecta: Hemiptera: Coccoidea), Fauna of New Zealand, No. 66 Lincoln Canterbury, New Zealand, 275 pp.

[B15] Henderson RC Sultan A Robertson AW (2010) Scale insect fauna (Hemiptera: Sternorrhyncha: Coccoidea) of New Zealand’s pygmy mistletoes (Korthalsella: Viscaceae) with description of three new species: *Leucaspisalbotecta*, *Leucaspistrilobata* (Diaspididae) and *Eriococcuskorthalsellae* (Eriococcidae) Zootaxa 2644: 1–24.

[B16] MacGillivray AD (1921) The Coccidae. Tables for the Identification of the Subfamilies and Some of the More Important Genera and Species, together with Discussions. Scarab, Urbana, Ill, 502 pp.

[B17] Mamet JR (1941) On some Coccidae (Hemipt. Homopt.) described from Mauritius by de Charmoy. Mauritius Institute Bulletin. Port Louis 2: 23–29.

[B18] Mille C Henderson RC Cazeres S Jourdan H (2016) Checklist of the scale insects (Hemiptera: Sternorrhyncha: Coccomorpha) of New Caledonia. Zoosystema 38: 129–176. 10.5252/z2016n2a1

[B19] Shimer H (1868) Notes on the “apple bark-louse” (*Lepidosaphesconchiformis* Gmelin sp.) with a description of a supposed new Acarus. Transactions of the American Entomological Society 1868: 361–374.

[B20] Takagi S (1969) Diaspididae of Taiwan based on material collected in connection with the Japan-U.S. Co-operative Science Programme, 1965 (Homoptera: Coccoidea). Part I. Insecta Matsumurana 32: 1–110.

[B21] Takagi S (1970) Diaspididae of Taiwan based on material collected in connection with the Japan-U.S. Cooperative Science Programme, 1965 (Homoptera: Coccoidea). Pt. II. Insecta Matsumurana 33: 1–146.

[B22] Takagi S (1999) For a better understanding of *Aulacaspis*: the Calcarata species group (Homoptera: Coccoidea: Diaspididae). Insecta Matsumurana 55: 133–180.

[B23] Williams DJ Miller DR Rung A (2006) A systematic revision of the armored scale genus *Furcaspis* Lindinger (Diaspididae: Coccoidea: Hemiptera). Contributions of the American Entomological Institute 34: 1–86.

[B24] Williams DJ Watson GW (1988) The Scale Insects of the Tropical South Pacific Region. Pt. 1. The Armoured Scales (Diaspididae). CAB International Wallingford, UK, 290 pp.

[B25] Wolff VRS (2001) Dez Espécies novas de *Pseudoparlatoria* Cockerell, 1892 (Hemiptera, Coccoidea, Diaspididae). Arquivos do Instituto Biológico São Paulo 68: 67–76.

[B26] Wolff VRS Claps LE (2008) Redescription of *Diaspidistis* Hempel, 1900 and new combinations of four *Pseudoparlatoria* species (Hemiptera: Coccoidea). Proceedings of the XI International Symposium on Scale Insect Studies, Oeiras, Portugal, 24–27 September 2007. ISA Press Lisbon, Portugal, 322 pp.

